# Cohort profile: the Swedish Prescribed Drugs and Health Cohort (SPREDH)

**DOI:** 10.1136/bmjopen-2018-023155

**Published:** 2019-01-28

**Authors:** Shao-Hua Xie, Giola Santoni, Fredrik Mattsson, Eivind Ness-Jensen, Jesper Lagergren

**Affiliations:** 1 Upper Gastrointestinal Surgery, Department of Molecular Medicine and Surgery, Karolinska Institutet, Karolinska University Hospital, Stockholm, Sweden; 2 HUNT Research Centre, Department of Public Health and Nursing, NTNU, Norwegian University of Science and Technology, Levanger, Norway; 3 Medical Department, Levanger Hospital, Nord-Trøndelag Hospital Trust, Levanger, Norway; 4 School of Cancer and Pharmaceutical Sciences, King’s College London, London, UK

**Keywords:** medications, cohort, register-based research, Sweden

## Abstract

**Purpose:**

The Swedish Prescribed Drugs and Health Cohort (SPREDH) is a Swedish population-based cohort based on data from four nationwide health data registers, created with the aim of investigating how the use of selected medications influences cancer risk and other outcomes.

**Participants:**

The cohort includes 8 421 115 users of selected common medications who have been followed-up for a total of 82 281 720 person-years from 1 July 2005 to 31 December 2015.

**Finding to date:**

The data in SPREDH were prospectively collected from the following national health data registers in Sweden: Prescribed Drug Register, Patient Register, Cancer Register and Causes of Death Register. Data on basic patient characteristics, use of the selected common medications, healthcare utilisation, diagnoses (including detailed information on cancers), and dates and causes of death are available for all cohort participants. The cohort currently includes 801 766 incident cancer cases.

**Future plans:**

The data in SPREDH can be used for various types of epidemiological research, particularly for examining how the use of the selected medications influences disease risk and other outcomes. We are initially planning cohort studies and nested case-control studies on selected medications in relation to the risk and prognosis of oesophageal and gastric cancers.

**Strengths and limitations of this study**

- The Swedish Prescribed Drugs and Health Cohort is population-based cohort based on data from four nationwide health data registers, with the aim of investigating the use of selected medications in relation to cancer risk and other outcomes.
- The follow-up of participants in the cohort is virtually complete and includes 82 281 720 person-years.
- Prospectively collected data on basic characteristics, use of selected medications, healthcare utilisation, diagnoses (including detailed information on all cancers), and dates and causes of death are available for all participants in the cohort.
- A weakness of the cohort is the lack of information on the use of medications before the starting date of the Prescribed Drug Register (1 July 2005) and lifestyle exposures.

## Introduction

Sweden might be regarded a paradise for epidemiological research because of the uniform healthcare system and the existence of nationwide population-based registers with long history and high completeness.^1 2^ The Swedish Prescribed Drug Register is a nationwide database of all prescribed and dispensed drugs, which provides excellent opportunities for pharmacoepidemiological research. The Swedish Prescribed Drug Register started in 1 July 2005 and contains information on over 100 million prescriptions per year.^3–5^ At least one prescribed drug has been purchased annually by approximately two-thirds of the population.^3^ Currently, this register contains information on a large number of prescriptions from the entire Swedish population since over 10 years ago. During the past decade, hundreds of pharmacoepidemiological studies have been published based on data from the Swedish Prescribed Drug Register, but these have mainly focused on drug utilisation patterns, safety and effectiveness.^3^ By linking data on individuals from the Swedish Prescribed Drug Register with other Swedish national health data registers, new opportunities emerge for research examining how the use of medications influences various health and disease outcomes.

Based on data from four nationwide Swedish health data registers, we have recently established a cohort of users of selected common medications, entitled the Swedish Prescribed Drugs and Health Cohort (SPREDH). This cohort aims to investigate the use of a broadly defined group of medications with sex hormonal effects mainly in relation to cancer risk. Because our research group is particularly interested in how sex hormonal exposures influence the risk of oesophageal and gastric cancers, for which the striking male predominance in incidence remains unexplained and recent data suggest that sex hormones may play a role,^6 7^ many of the selected medications are known to have effects on sex hormone levels and fertility. For comparison purposes, the cohort also includes users of some other commonly prescribed drugs which are used for similar medical indications as medications with sex hormonal effects. Because of the large number of variables collected from multiple health data registers, the cohort can also be used to investigate various health and disease outcomes other than cancer.

## Cohort description

### Source data

The data in SPREDH were collected from the following four national health data registers in Sweden: the Prescribed Drug Register, Patient Register, Cancer Register and the Causes of Death Register. All these registers have nationwide complete coverage and provide high quality data. Information on an individual person from different registries can be linked by means of the 10-digit unique personal identity number assigned to each Swedish resident from birth or immigration.^8^ Here, we only briefly describe these registries. More detailed information can be found elsewhere.^3 4 9–11^

*The Swedish Prescribed Drug Register* was established on 1 July 2005 and contains patient-level data on all drugs prescribed and dispensed in outpatient care in the entire Swedish population.^3 4^ All drugs are classified according to the Anatomical Therapeutic Chemical classification system and defined daily doses are used for the measurement of drug utilisation. Information on dispensed prescriptions mainly includes date of prescribing and dispensing, dispensed item (substance, brand name, formulation and package size), dispensed amount and dosage. Basic patient information includes age, sex, place of residence and personal identity number. The Swedish Prescribed Drug Register does not include data on over-the-counter medications or drugs used only in hospitals. It has been estimated that prescribed drugs in the Swedish Prescribed Drug Register account for 84% of the total drug sales in Sweden.^3 4^

*The Swedish Patient Register* covers all inpatient care in Sweden since 1987 and all specialised outpatient care from both public and private sectors since 2001. Information available in the Patient Register mainly includes patient-related data (eg, age, sex and personal identity number), administrative data (eg, dates of admission and discharge), diagnoses and surgical procedures. The diagnoses are coded according to the Swedish versions of the International Classification of Disease (ICD) system.^10^ The Patient Register has almost 100% complete recording of all inpatient care. The coverage of specialised outpatient care is also almost 100% complete for data from public caregivers, but the overall completeness is lower (80%) due to missing data from private caregivers.^10^

*The Swedish Cancer Register* started on a nationwide level in 1958 and the overall completeness has been estimated as approximately 96%.^11^ Information in the Cancer Register includes data on the patient (eg, sex, age, place of residence at diagnosis and the personal identity number), date of diagnosis, basis of diagnosis, anatomic site and histological type of the tumour and tumour stage. Sites of malignancies have been coded according to different versions of ICD system over time in the Swedish Cancer Register; however, codes according to the seventh version of ICD (ICD-7) are available for the whole registration period from 1958 onwards. The registration of basal cell carcinoma in Sweden started in 2003, and the complete data covering the whole population are available in the Swedish Cancer Register from 2004 onwards.^12^

*The Swedish Causes of Death Register* covers all deaths of persons who were registered in Sweden in the year they died since 1952 and has a 100% complete coverage for date of death.^9^ This register also includes patient-related data (eg, age, sex, place of residence and personal identity number). As well as the date of death, both the underlying cause (the primary cause) and contributing causes (secondary causes) of death are recorded. Among all individuals in the Causes of Death Register, 96% have a specific underlying cause of death recorded.^9^

### Participants

All individuals who had purchased any drug included in the groups of medications listed in table 1 from 1 July 2005 to 31 December 2015, as recorded in the Swedish Prescribed Drug Register, were included in SPREDH. The selected medications include the three following categories. First, we included medications which are known antagonists and modulators of sex hormones and widely used to treat sex hormone-related disorders or diseases in sex hormone dependent organs, that is, (1) sex hormones and modulators of the genital system, (2) drugs used against benign prostatic hypertrophy and (3) hormones, hormone antagonists, and related agents in antineoplastic and immuno-modulating therapy. Second, we selected a group of medications not usually prescribed for treating sex hormone related disease, but known to have substantial effects on sex hormone levels and fertility, including (1) metformin,^13^ (2) spironolactone,^14^ (3) statins,^15^ (4) non-steroidal anti-inflammatory drugs^16^ and (5) H2-receptor antagonists.^17^ Third, for comparison purposes, we also included drugs for similar medical indications as the second category, but without any influence on sex hormone levels, that is, (1) medications in the treatment of diabetes other than metformin, (2) diuretics other than spironolactone, (3) lipid modifying agents other than statins, (4) other anti-inflammatory agents, analgesics and platelet aggregation inhibitors and (5) other drugs for peptic ulcer and gastro-oesophageal reflux disease. The use of the comparison groups (the third category) is scientifically crucial because it counteracts information bias from patients receiving more medical attention due to medication and bias from confounding by any factors associated with the indication for the medication. All participants have been followed-up from the date of their first known purchase of any of the listed medications until the dates of death (through linkage to the Causes of Death Register) or the end of study period (31 December 2015), whichever occurred first.

**Table 1:** Medication groups included in this study and their Anatomical Therapeutic Chemical (ATC) classification codes

Medications	ATC codes
Sex hormones and modulators of the genital system	G03
Metformin and other medications for the treatment of diabetes	A10
Drugs used against benign prostatic hypertrophy	G04C
Hormones, hormone antagonists, and related agents in antineoplastic and immunomodulating therapy	L02
Spironolactone and other diuretics	C03
Statins and other lipid modifying agents	C10
Non-steroidal anti-inflammatory drugs and other anti-inflammatory agents/analgesics/ platelet aggregation inhibitors	M01, N02, B01AC
H2-receptor antagonists and other drugs for peptic ulcer and gastro-oesophageal reflux disease	A02B, J01CA04, J01FA, J01XD, J01AA, J01MA, J01XE, J04AB04

### Variables

**Figure 1:**
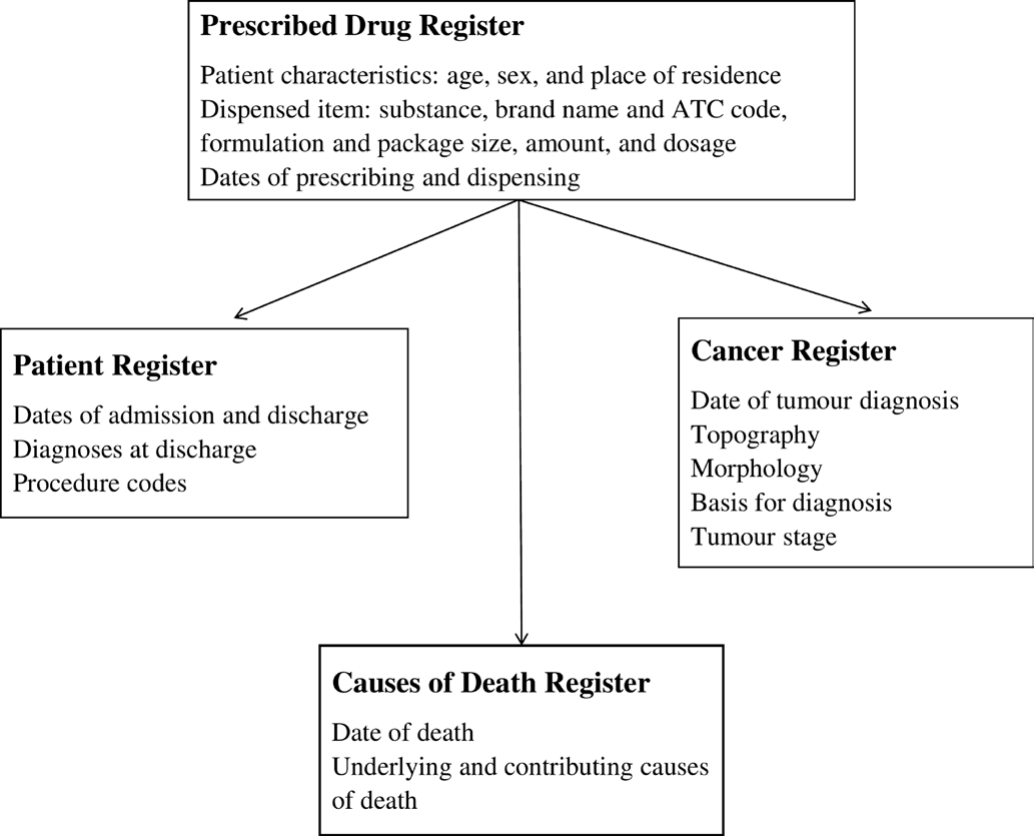
Registers from which data for the Swedish Prescribed Drugs and Health Cohort have been retrieved and major variables included. ACT, Anatomical Therapeutic Chemical

Prospectively collected data on basic patient characteristics, use of the selected common medications, healthcare utilisation, diagnoses (including detailed information on cancers) and dates and causes of death are available for all cohort participants. The data are available for all participants from the earliest possible date since the start of each register until 31 December 2015 and include all variables listed above for each register. Major variables obtained from each register and included in the SPREDH cohort are presented in figure 1.

### Ethical approval

Separate ethical approval may be required for studies involving other research questions. Register-based research is exempt from informed consent from individual participants in Sweden.^18^

### Patient and public involvement

There was no patient or public involvement in this study.

## Findings to date

The cohort currently consists of 8 421 115 participants who have been followed-up for a total of 82 281 720 person-years. The cohort includes slightly more female (54.4%) participants than males (45.6%), with a wide range of age distribution (from below 20 to over 65 years) at entry. Among all participants, 4 652 031 were recruited in 2005, corresponding to 51% of the total population (9.4 million) in Sweden in this year. Some characteristics of the cohort participants at entry are presented in table 2.

**Table 2:** Characteristics of participants in the Swedish Prescribed Drugs and Health Cohort

	Number of participants (%)	Person-years
Total	8 421 115 (100.0)	82 281 720
Age at entry years		
0–19	1 748 819 (20.8)	16 732 539
20–44	2 748 489 (32.7)	27 953 877
45–64	2 301 156 (27.3)	24 138 640
65+	1 620 458 (19.2)	13 456 664
Sex		
Male	3 839 432 (45.6)	36 497 277
Female	4 579 490 (54.4)	45 784 443
Calendar year of entry		
2005	4 652 031 (55.2)	49 242 254
2006	1 500 691 (17.8)	16 246 080
2007	690 175 (8.2)	6 783 055
2008	420 363 (5.0)	3 712 928
2009	285 558 (3.4)	2 238 964
2010–2015	872 297 (10.4)	4 058 414

Until 31 December 2015, SPREDH includes 787 776 incident cancer patients, among which including 86 405 with breast cancer (85 965 females and 440 males), 82 923 with uterine cancer, 10 263 with ovarian cancer, 101 724 with prostate cancer and 3278 with testicular cancer (table 3). There are clearly more male cases of oesophageal and gastric cancers than females, particularly for oesophageal adenocarcinoma with as high as 82.1% of all cases in males (table 4). The age distribution of cancers in sex hormone dependent organs is presented in table 5. Overall, the large number of cancer cases in SPREDH would provide adequate statistical power for many research questions in cancer research, particularly regarding common cancers.

**Table 3:** Number of incident cases of malignancies according to the seventh edition of the International Classification of Diseases (ICD-7) in the Swedish Prescribed Drugs and Health Cohort until 31st December 2015

Sites	ICD-7 codes	Number of cases (%)
Lip, oral cavity and pharynx	140–148	11 436 (1.1)
Digestive organs	150–157	122 693 (15.6)
Oesophagus	150	4782 (0.6)
Stomach	151	9496 (1.2)
Colon, rectum and anus	153–154	83 857 (10.6)
Liver and biliary tract	155	9533 (1.2)
Pancreas	157	11 140 (1.4)
Lung (including trachea and bronchus)	162	40 315 (5.1)
Breast	170	86 405 (11.0)
Uterus	171, 172, 174	82 923 (10.5)
Ovary	175	10 263 (1.3)
Prostate	177	101 724 (12.9)
Testis	178	3278 (0.4)
Bladder	181	25 835 (3.3)
Kidney	180	12 754 (1.6)
Thyroid	194	5041 (0.6)
Hodgkin lymphoma	201	2149 (0.3)
Non-Hodgkin’s lymphoma	200, 202	18 971 (2.4)
Leukaemia	204–207	16 310 (2.1)
All	140–209	787 776 (100.0)

**Table 4:** Number (%) of incident cases of oesophageal and gastric cancers by sex in the Swedish Prescribed Drugs and Health Cohort until 31st December 2015

Cancer type	Total	Men	Women
Oesophageal adenocarcinoma	2396 (100.0)	1968 (82.1)	428 (17.9)
Oesophageal squamous cell carcinoma	1770 (100.0)	1076 (60.7)	694 (39.2)
Gastric adenocarcinoma	7556 (100.0)	4675 (61.9)	2881 (38.1)

**Table 5:** Number (%) of incident cases of malignancies in sex hormone dependent organs by age in the Swedish Prescribed Drugs and Health Cohort until 31st December 2015

Sites	Total	Age, years				
		<50	50–59	60–69	70–79	80+
Breast	86 405 (100.0)	15 620 (18.1)	16 858 (19.5)	25 318 (29.3)	16 349 (18.9)	12 260 (14.2)
Breast (only females)	85 965 (100.0)	15 581 (18.1)	16 792 (19.5)	25 200 (29.3)	16 233 (18.9)	12 159 (14.1)
Cervix uteri	66 212 (100.0)	60 212 (90.9)	3265 (4.9)	1413 (2.1)	790 (1.2)	593 (0.9)
Corpus uteri	15 342 (100.0)	1131 (7.4)	2436 (15.9)	4563 (29.7)	4286 (27.9)	2926 (19.1)
Ovary	10 263 (100.0)	2348 (22.9)	2024 (19.7)	2791 (27.2)	1976 (19.3)	1124 (11.0)
Prostate	101 724 (100.0)	929 (0.9)	11 939 (11.7)	41 092 (40.4)	32 406 (31.9)	15 358 (15.1)
Testis	3278 (100.0)	2832 (86.4)	260 (7.9)	100 (3.1)	63 (1.9)	23 (0.7)

### Future plans

The data available in SPREDH can be used for various types of epidemiological research. First, the data can be used for monitoring trends and patterns of drug utilisation, which can serve as indicators for disease occurrence and treatment efficacy.^19 20^ For example, use of anti-reflux medication (mainly proton pump inhibitors) can provide an estimate of the prevalence of gastro-oesophageal reflux disease in the population, while decreased use of anti-reflux medication in reflux patients may indicate decreased gastro-oesophageal reflux symptoms. Second, cohort studies or nested case-control studies can be carried out to evaluate how the use of specific medications per se influences disease risk and other outcomes. We are planning a nested case-control study to examine how the use of angiotensin-converting enzyme inhibitors, statins and non-steroidal anti-inflammatory drugs influences the risk of progression from Barrett’s oesophagus to oesophageal adenocarcinoma. We will identify all incident cases of oesophageal adenocarcinoma during follow-up in SPREDH and randomly select a comparison group of cancer-free patients with Barrett’s oesophagus using an incidence density sampling strategy. We will then compare the use of medications listed above in these two groups and adjust the results for potential confounders, including comorbidities and use of other relevant medications. In another cohort study, we will examine how the use of non-steroidal anti-inflammatory drugs influences the prognosis in patients diagnosed with stomach and oesophageal cancers. In addition to these example studies and a few others that are already planned and are within our main research interests, the data in SPREDH can be used to answer many other research questions.

## Strengths and weaknesses

Strengths of SPREDH include the large sample size (the majority of the entire Swedish population), complete follow-up, good quality data with nationwide coverage and a large number of variables, which enable investigations of various health and disease outcomes. Moreover, by using the unique personal identity number, there are also good possibilities to link SPREDH to additional registers and databases for obtaining further information when necessary.^8^

A weakness of SPREDH is the lack of information on the use of medications before the starting date of the Prescribed Drug Register, that is, 1 July 2005. For example, in studies examining how use of a medication influences a given outcome, there will be certain exposure misclassification due to missing information before this date. However, because such exposure misclassification would have affected all cohort members similarly, regardless of the outcome of interest, this bias is probably non-differential (independent of the outcome) and would thus result in underestimated associations between exposures and outcomes rather than explaining them. In addition, because a prescription is valid for a maximum of 12 months in Sweden, it is possible to define an ‘incident’ exposed group of participants by excluding those with a first known prescription in the first year of the cohort establishment and explore the exposure-response association in these ‘incident’ exposed participants.^21 22^ Although the lack of data on medication use before 2005 challenges whether these ‘incident’ users are first-time users or re-starters, linkage to other registers may provide useful information, for example, date of first diagnosis as recorded in the Patient Register (initiated in 1965 and nationwide complete from 1987), for selecting study participants or conducing sensitivity analyses. However, potential users of the data should be aware of that data from specialised outpatient care in the Patient Register were only available from 2001 onwards and primary care is not covered. Thus, the validity of using data from the Patient Register will depend on the specific research question. A ‘washout period’ of the first year of the Prescribed Drug Register may be used to exclude prior use of a medication in order to identify ‘current users’ and ‘re-initiators’ among those who had stopped but resumed use later.^22^ Another major limitation is residual and unmeasured confounding due to a lack of data on lifestyle exposures, including tobacco smoking, alcohol use and dietary factors.^2 23^ Potential solutions to this problem include the use of proxy measures for relevant confounders (eg, smoking-related and alcohol-related conditions and diseases), external or indirect adjustment, sensitivity analyses and the use of negative control outcomes.^24–26^ Additional data collected through surveys, quality registers and clinical cohorts in Sweden can be linked to SPREDH by using the unique personal identity number, but they would only be available for a small subset of the cohort. Although the cohort covers the majority of the Swedish population, all participants have had at least one dispensing of any of the selected medications from a pharmacy and thus represent a subset of the general population seeking care for certain medical conditions. This selection means that future research findings may be interpreted with caution, depending on the specific research question to be investigated. In addition, the follow-up is relatively short (maximum 10 years), which reduces the statistical power and limits studies requiring a long time latency period between the exposure and the outcome. Therefore, future updates of SPREDH with additional years of follow-up will be useful and we will plan to update the cohort with new data and longer follow-up every few years in the future.

## Collaboration

All data from SPREDH presented in this article are stored by the research group of the authors on safe servers at Karolinska Institutet, Sweden and handled confidentially. Currently, only the research team has access to the data. Researchers interested in collaboration are welcome to contact Professor Jesper Lagergren (jesper.lagergren@ki.se), principal investigator or Assistant Professor Shao-Hua Xie (shaohua.xie@ki.se), co-investigator.
